# Fatal Impaction of the Bowels from Cheese

**Published:** 1883-05

**Authors:** Charles W. Miller

**Affiliations:** Peabody, Kansas


Fatal Impaction of the Bowels from Cheese. By Charles W. Miller, M. D., Peabody, Kansas.

One evening about dusk, in the latter part of Februrary, 1879, I was hastily summoned from my office by a Russian farmer living fifteen miles northwest of this city, in the interest of a sick neighbor of his by the name of Abraham Base, also a Russian, whom he represented, as well as I could determine, as suffering greatly from flatulent colic. Supposing, from what I could understand from the Russian's description of the case, that it was nothing more than an ordinary case of colic, induced probably by over eating or by the use of very improper food, I merely supplied myself with a good supply of ipecacuanha and morphia, and started on my mission in company with my Russian friend on a journey of fifteen miles of lonely prairie, during one of the coldest nights of that intensely cold winter. Arriving at one of those peculiar Russian houses, consisting of mud and straw—the mud in the form of Russian brick, from which the house is constructed, and the straw for the thatched roof overhead—I found myself among a group of solemn, religious people, who were anxiously awaiting my arrival. My patient was lying upon his back in bed, with his legs drawn up, his knees almost touching his abdomen, in agony indescribable, the sweat pouring down over his face, and with all of his undergarments as wet as though he had just been lifted out of a hot bath. He was a tall, heavy man, muscularly built and powerfully constituted, somewhere in the neighborhood of thirty years of age; married, with wife and family of small children surrounding him. Seeing at a glance that I had no ordinary case to deal with, and that the diagnosis I had anticipated was very probably a visionary one after all, I commenced to probe the family for a history of the case in the best German I could command, while seemingly examining his tongue and pulse. The results were that he had been confined to the house with abdominal pains three or four days previously, the pains increasing in severity each successive day; that they came about in the first instance from a vist to Newton, a town distant about fourteen miles, which he had made some days previously in a large, heavy, jolting farm-wagon, eating about a pound and a half of store cheese on his homeward journey; the jolting, bumping movement of the wagon packing it down for him *solidly* about as fast as it reached the small intestines; this destination being reached also somewhat faster than ordinary by the rough manner of the journey. He was now suffering from peritonitis, with all of its concomitant symptoms; vomiting of fæcal matters; dry, parched condition of the tongue; extremely foul breath; stercoraceous vomiting; partial coma; extremely swift and feeble pulse; deeply injected eyes; flatulent condition of lower bowels; the impaction being marked and fairly outlined against the abdomen. There had been no stools for twenty-four hours previously; nothing passing but flatus, which was frequent and involuntary. No retention whatever, by mouth or rectum, of anything solid or liquid, in quantities however small. Seeing, therefore, at once the hopeless situation of my patient as well as myself, I concluded to send for my partner for consultation, in the meantime employing message gently and persistently over the seat of impaction, in the desperate hope of moving the mass.

We labored earnestly until dawn by the side of the doomed Russian, doing all in our power to move the mass, but nothing came but flatus. An operation would have been useless at the advanced stage in which the case was first presented to us, as well as firmly objected to by a people believing religiously in the will of God in all such matters. Singular to relate, the man lived nearly forty-eight hours afterwards, dying only when mortification had long existed to a great extent over the seat of impaction. —*Southern Clinic.*

